# Arthroscopic Anterior Cruciate Ligament Repair Versus Autograft Anterior Cruciate Ligament Reconstruction: A Meta-Analysis of Comparative Studies

**DOI:** 10.3389/fsurg.2022.887522

**Published:** 2022-04-20

**Authors:** Long Pang, Pengcheng Li, Tao Li, Yinghao Li, Jing Zhu, Xin Tang

**Affiliations:** ^1^ Department of Orthopedics, Orthopedic Research Institute, West China Hospital, Sichuan University, Chengdu, China; ^2^ Department of Respiratory and Critical Care Medicine, West China Hospital, Sichuan University, Chengdu, China

**Keywords:** ACL (anterior cruciate ligament), arthroscopic ACL repair, autograft ACL reconstruction, meta-analysis, arthroscope

## Abstract

**Purpose:**

To compare the clinical outcomes of arthroscopic anterior cruciate ligament (ACL) repair and autograft ACL reconstruction for ACL ruptures.

**Methods:**

PubMed, EMBASE, Scopus, Web of Science and The Cochrane Library were searched for relevant studies from 1 January 1990 to 21 March 2022. Two evaluators independently screened the literature, extracted data and assessed the methodological quality of the enrolled studies. Meta-analysis was conducted using RevMan 5.4 software.

**Results:**

Ten studies with mean follow-up periods from 12 to 36 months were included. For 638 patients with ACL ruptures, arthroscopic ACL repair showed statistically comparable outcomes of failure (*p* = 0.18), complications (*p* = 0.29), reoperation other than revision (*p* = 0.78), Lysholm score (*p* = 0.78), Tegner score (*p* = 0.70), and satisfaction (*p* = 0.45) when compared with autograft ACL reconstruction. A significantly higher rate of hardware removal (*p* = 0.0008) but greater International Knee Documentation Committee (IKDC) score (*p* = 0.009) were found in the ACL repair group. The heterogeneity of the side-to-side difference of anterior tibial translation (ΔATT) was high (*I*^2 ^= 80%). After the sensitivity analysis, the *I*^2^ decreased dramatically (*I*^2 ^= 32%), and the knees with ACL repair showed significantly greater ΔATT (*P* = 0.04).

**Conclusion:**

For proximal ACL ruptures, arthroscopic ACL repair showed similar clinical outcomes, and even better functional performance when compared to autograft ACL reconstruction. ACL repair has a higher rate of hardware removal, and might be related to greater asymptomatic knee laxity. More high-quality prospective trials are needed to confirm our findings.

## Introduction

Primary open repair of the anterior cruciate ligament (ACL) was the standard surgical technique for ACL ruptures until the 1980s, when poor results at longer follow-up were reported (1–4). Thereafter, open ACL repair was gradually replaced by arthroscopic autograft ACL reconstruction for ACL ruptures.

However, autograft ACL reconstruction has several problems, such as anterior knee pain, thigh muscle weakness following harvesting, and an extensive rehabilitation period (5). In the past decade, renewed and increasing interest in ACL repair has arisen and various arthroscopic ACL repair techniques have been introduced, especially for proximal ACL ruptures (6, 7). Among these newly developed surgeries, four ACL repair techniques have been in the spotlight (5): suture anchor repair (SAR) of the ACL, repair with dynamic intraligamentary stabilization (DIS), internal brace ligament augmentation (IBLA) and bridge-enhanced ACL repair (BEAR). Moreover, previous published systematic reviews showed promising results following ACL repair. In 2017, Ahmad et al. (7) analyzed 23 articles related to DIS and concluded that ACL repair might be an effective modality for the treatment of acute proximal ACL tears. In another systematic review, van der List et al. (8) found that different techniques of primary ACL repair were safe with failure rates between 7 and 11%, and good functional outcome scores in 1,101 patients. However, subsequent two systematic reviews (9, 10) reported inconsistent results following ACL repair compared with ACL reconstruction.

On further review of the above literatures (7–10), we found most included studies in these systematic reviews were case series with relatively small sample sizes, and the number of high-quality trials comparing arthroscopic ACL repair with reconstruction was scarce, making it difficult to conduct a meta-analysis comparing arthroscopic ACL repair with autograft ACL reconstruction. Importantly, there are several high-quality comparative clinical trials (11–14) published in recent years reporting that arthroscopic ACL repair yields ideal results comparable to those of ACL reconstruction. Therefore, the purpose of this meta-analysis was to quantitatively compare the clinical outcomes of arthroscopic ACL repair and autograft ACL reconstruction for ACL ruptures. The study is to explore that whether arthroscopic ACL repair can be a viable alternative to ACL reconstruction for patients with ACL ruptures, providing an optional surgery for treating ACL tears in clinical practice.

## Methods

### Literature Search

This study was conducted in strict accordance with the Preferred Reporting Items for Systematic Reviews and Meta-Analyses (PRISMA) guidelines (15). We searched PubMed, Embase, Scopus, Web of Science, and the Cochrane Library, using the combination of “anterior cruciate ligament”, “ACL”, “repair”, “suture”, “dynamic intraligamentary stabilization”, “internal brace”, “bridge-enhanced”, “autograft” and “reconstruction”. The search was performed by two independent researchers. All possible studies from 1 January 1990 to 21 March 2022, were manually retrieved. In addition, the reference lists of all retrieved articles were reviewed for potentially eligible studies. Any disagreement was debated and resolved with a third researcher.

### Inclusion and Exclusion Criteria

Inclusion criteria: (1) the enrolled patients had a confirmed diagnosis of ACL rupture; (2) clinical studies comparing primary arthroscopic ACL repair to autograft ACL reconstruction; (3) a minimum of 12-month follow-up; (4) written in English.

Exclusion criteria: (1) presence of multiligamentous injuries or knee dislocations; (2) presence of concomitant lesions that would alter the rehabilitation; (3) presence of previous knee injuries on either the injured or contralateral knee; (4) long-term follow-up of historical studies; (5) focused on skeletally immature patients; (6) study with smallest cohort or shortest follow-up (different studies that report on the same group of patients); (7) data inadequate or unavailable.

### Data Extraction

Two researchers independently extracted data from the included studies. Characteristics of the enrolled studies (authors, year, country, study design, level of evidence, sample size), and patients’ baseline information (age, sex, time from injury to surgery, follow-up period, rupture location, autograft). The following clinical outcomes were extracted and pooled: (1) failure, complications, reoperation other than revision, hardware removal rates; (2) anteroposterior (AP) knee laxity assessed by the mean difference in anterior tibial translation (ΔATT) between the injured and contralateral knees; and (3) patient-reported outcomes including the International Knee Documentation Committee (IKDC) score (16), the Lysholm score (17), the Tegner score (18), and satisfaction.

### Quality Assessment

Two authors made their own assessment of the risk of bias of enrolled randomized controlled trials (RCTs) according to the Risk of Bias Tool conferred by the Cochrane Handbook (19), and those of cohort and case–control studies by the Methodological Index for Non-Randomized Studies (MINORS) criteria (20). A third evaluator made the final decision when any disagreements appeared.

### Statistical Analysis

Statistical analyses were performed with Manager V.5.4 (The Cochrane Collaboration, Software Update, Oxford, UK). We analyzed the outcomes by calculating the weighted mean difference (WMD) and pooled odds ratio (OR) with corresponding 95% confidence intervals (CIs). A *P* value <0.05 was considered to be statistically significant. We evaluated and characterized the heterogeneity of each eligible study with Cochrane’s Q statistic and *I*^2^ statistics. Statistical heterogeneity between studies was assessed using the *I*^2^ value, where less than 50% was considered to be within the acceptable range of heterogeneity and the fixed effect model was applied. Otherwise, the random effect model was adopted. In case of any heterogeneity, the following methods were applied to explain: (1) sensitivity analysis; (2) subgroup analysis. Sensitivity analyses were conducted to confirm the robustness of pooled outcomes by sequentially removing the included studies one-by-one. Subgroup analyses according to RCT design or non-RCT design were conducted for all outcomes. For the primary outcome failure rates, subgroup analyses according to the rupture location and ACL repair technique were performed. Publication bias was not assessed because the number of studies included in each study area was less than 10; therefore, the statistical power was low.

## Results

### Literature Search

**Figure 1:**
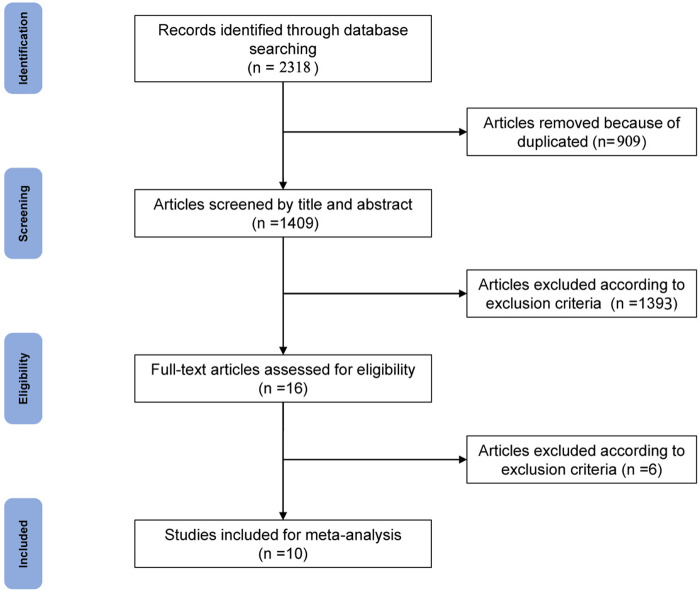
PRISMA flow chart of literature retrieval.

Two independent researchers searched PubMed, Embase, Scopus, Web of Science and the Cochrane Library according to the identified keywords, and a total of 2,318 articles were retrieved. After 909 duplicates were removed, the titles and abstracts of 1,409 remaining articles were screened, and 1,393 articles were removed according to the inclusion and exclusion criteria. The remaining full texts of 16 articles were screened independently by two researchers. The study by Gagliardi et al. (21) was excluded because this cohort enrolled skeletally immature patients (7–18 years old). The study by Jonkergouw et al. (22) was excluded because it compared outcomes following ACL repair with or without additional internal bracing. The study by Sporsheim et al. (23) was excluded because it was a 30-year follow-up of a history study, in which the contemporary arthroscopic ACL repair technique was not performed. The study by van der List et al. (24) was excluded because the follow-up period was less than 12 months. The study by Connolly et al. (25) excluded only reported postoperative pain and narcotic prescriptions at the first visit. Two studies (26, 27) by Murray et al. focused on the same group of patients, and the study with shorter follow-up (27) was excluded. Finally, 10 articles (11–14, 26, 28–32) were included in the meta-analysis, including 4 RCTs (11–14), 1 prospective cohort study (26), 3 retrospective cohort studies (28, 29, 31), and 2 case–control studies (30, 32) (Figure 1).

### Quality Assessment

**Figure 2:**
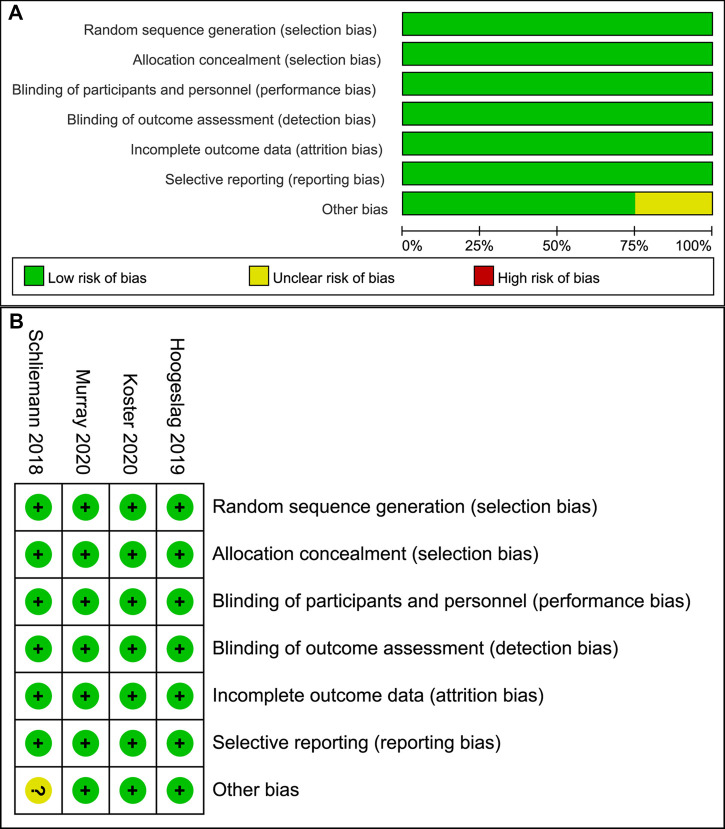
Risk of bias graph (**A**) Graph of the risk of bias for the included RCTs; (**B**) graph of the risk of bias summary for the included RCTs.

The quality of the included RCTs was assessed using Review Manager software, and they are summarized and visualized in Figure 2. The quality of the included cohort and case–control studies was evaluated by MINORS criteria, which are shown in Table 1.

**Table 1:** Quality assessment of the non-RCT studies using the Methodological Index for Non-Randomized Studies (MINORS) criteria.

Author	Year	Journal	Study design	LOE	1	2	3	4	5	6	7	8	Total
Achtnich	2016	Arthroscopy	Case-control	III	2	2	1	2	1	2	2	0	12
Bieri	2017	Injury	Case-control	III	2	2	2	2	1	2	2	0	13
Murray	2019	OJSM	PCS	II	2	2	2	2	2	2	2	1	15
Vermeijden	2020	Arthroscopy	RCS	III	2	2	2	2	1	2	2	0	13
Ortmaier	2021	Sportverletz Sportschaden	RCS	III	2	2	1	2	1	2	1	0	11
Szwedowski	2021	J Clin Med	RCS	III	2	2	1	2	1	2	2	2	14

*PCS, prospective cohort study; RCS, retrospective cohort study; LOE, level of evidence.*

*Only the non-comparative part of the MINORS criteria was used (i.e. first 8 questions). The criteria of MINORS with 0 points when not reported, 1 when reported but not adequate, and 2 when reported and adequate. Maximum score is 16.*

*1. A clearly stated aim; 2. Inclusion of consecutive patients; 3. Prospective collection of data; 4. End points appropriate to the aim of the study; 5. Unbiased assessment of the study end point; 6. Follow-up period appropriate to the aim of the study; 7. Loss to follow-up less than 5%; 8. Prospective calculation of the study size.*

### Baseline Data

Detailed baseline information for all included articles is summarized in Table 2, with a total of 638 patients assigned to the two groups (ACL repair/ACL reconstruction = 330/308). The mean age of patients ranged from 17 to 36 years old, and the percentage of male patients was from 30 to 81%. The mean time from injury to surgery ranged from 13 to 74 days, and the follow period ranged from 12 to 36 months. Two studies (14, 30) enrolled patients independent of the ACL rupture location, while other studies (11–13, 26, 28, 29, 31, 32) mainly included patients with ACL proximal rupture. As for the selection of autograft for ACL reconstruction, most studies (11–14, 26, 28, 32) used semitendinosus tendon, while the other studies (29–31) used hamstring tendon, patellar tendon, or quadriceps tendon. For the ACL repair techniques, primary SAR of the ACL was performed in 1 study (32), using one suture anchor to reattach the ACL remnant back to the femoral footprint. A total of 4 studies (11, 12, 14, 30) reported outcomes following primary ACL suture repair with DIS. After the remaining threads in the ACL stump were tensioned and the tibial stump was repositioned to the femoral footprint, an intraligamentary braid with cortical button fixation on the femoral side and an additional elastic link (a spring-in-screw mechanism) on the tibial side was introduced. A total of 2 studies (13, 26) by Murray et al. focused on patients treated with BEAR, involving suture repair of the ligament combined with a BEAR scaffold saturated with 5–10 ml autologous blood to bridge the gap between the ends of the torn ligament. In the other 3 studies (28, 29, 31), after completion of ACL suture repair, IBLA was achieved by adding FiberTape to the ACL substance, and fixed with a suture anchor perpendicular to the tibial cortex.

**Table 2:** Characteristics of the included studies.

Author	Year	Country	Study design	LOE	Sample size, n		Age, y Mean ± SD (Range)		Sex (M/F)		Time from injury to surgery, d Mean ± SD (Range)		Follow-up period, m Mean ± SD (Range)	Rupture location		Autograft
					Repair	Recon	Repair	Recon	Repair	Recon	Repair	Recon		Repair	Recon	
**Suture anchor repair (SAR)**																
Achtnich	2016	Germany	Case-control	III	20	20	30 ± 8.9	33.6 ± 3.7	NA	NA	<42	<42	28 ± ─ (24–31)	Proximal rupture		Semitendinosus tendon
**Dynamic intraligamentary stabilization (DIS)**																
Bieri	2017	Switzerland	Case-control	III	53	53	30 ± 8.5	31 ± 7.6	43/10	43/10	14 ± 12.8	50 ± 27.3	24	Independent		Hamstring tendon (67%), patellar tendon (27%), quadriceps tendon (6%)
Schliemann	2018	Germany	RCT	I	30	30	28.2 ± 11.4	29.1 ± 12.0	15/15	8/22	15.2 ± 4.5	16.3 ± 5.0	12	Independent		Semitendinosus tendon
Hoogeslag	2019	Netherlands	RCT	I	24	24	21 ± ─ (10–27)	22 ± ─ (19.3–25)	19/5	18/6	13 ± ─ (12–16)	47 ± ─ (42–71)	24	Proximal:83.3% Central:12.5% Distal:4.2%	Proximal: ─ Central: ─ Distal: ─	Semitendinosus tendon
Kosters	2020	Germany	RCT	I	43	42	28.7 ± 11.4	27.6 ± 10.6	25/18	31/11	14.5 ± 5.2	16.2 ± 7.3	24	Proximal:90.7% Midsubstance:9.3%	Proximal:76.2% Midsubstance:23.8%	Semitendinosus tendon
**Bridge-enhanced ACL repair (BEAR)**																
Murray	2019	USA	PCS	II	10	10	24.1 ± 4.9 (18.1–34.6)	24.6 ± 5.5 (18.6–33.8)	4/6	2/8	20.8 ± 4.8 (11.0–28.0)	24.6 ± 5.5 (24.0–80.0)	24	Length of tibial remnant, % 50–74: 90% 75–100:10%	Length of tibial remnant, % 50–74: 60% 75–100:40%	Semitendinosus-gracilis tendon
Murray	2020	USA	RCT	I	65	35	17 ± 1	17 ± 2	28/37	16/19	36 ± ─ (29–42)	39 ± ─ (33–43)	24	Length of tibial remnant, % 50–74: 88% 75–100:12%	Length of tibial remnant, % 50–74: 80% 75–100:20%	Semitendinosus-gracilis tendon (*n* = 33), bone–patellar tendon–bone autograft (*n* = 2)
**Internal brace ligament augmentation (IBLA)**																
Vermeijden	2020	USA	RCS	III	49	34	34.4 ± 10.7	29.4 ± 11.1	24/25	20/14	36 ± ─ (17–86)	74 ± ─ (34–186)	30 ± 9.6/36 ± 13.2	Proximal rupture		Soft-tissue allograft (*n* = 14), bone-patellar tendon-bone autograft (*n* = 9), hamstring autograft (*n* = 4), hybrid graft (*n* = 3)
Ortmaier	2021	Germany	RCS	III	24	45	NA	NA	8/16	16/29	<21	<21	21	Proximal rupture		Hamstring tendon (*n* = 25) quadriceps tendon (*n* = 20)
Szwedowski	2021	Poland	RCS	III	12	15	36 ± ─ (15–55)	NA	7/5	NA	30–60	30–60	14.8 ± ─ (5–24)/ 13.6 ± ─ (10–24)	Proximal rupture		Semitendinosus -gracilis tendon

*RCT, randomized controlled trial; PCS, prospective cohort study; RCS, retrospective cohort study; LOE, level of evidence; Recon, reconstruction; M, male; F, female.*

### Failure, Complications, Reoperation Other than Revision, and Hardware Removal Rates

**Figure 3:**
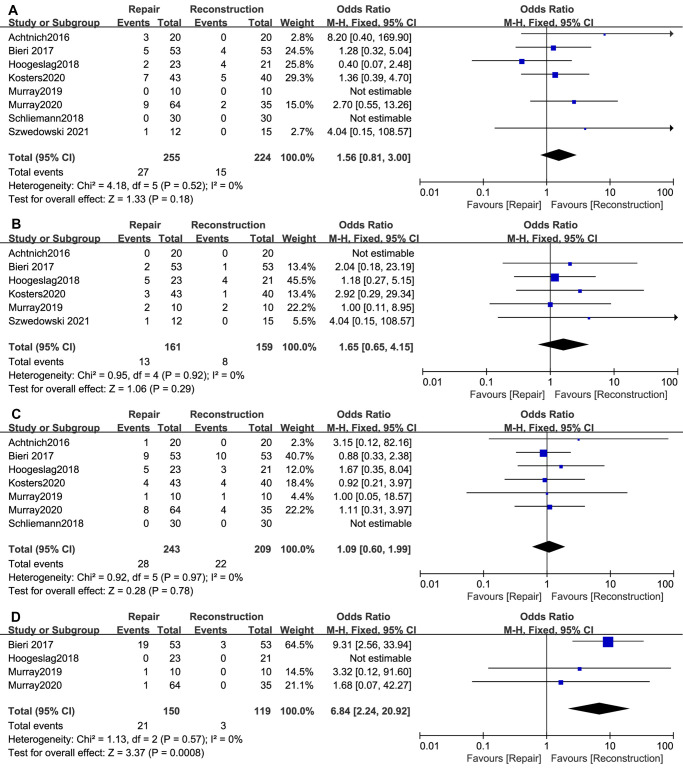
Meta-analysis of the rates of (**A**) Failure; (**B**) Complications; (**C**) Reoperation rather than revision; (**D**) Hardware removal.

Failures were defined as postoperative recurrent instability of the ipsilateral knee joint regardless of the need for revision surgery. A total of 9 studies (11–14, 26, 28, 30, 32) reported failures after surgery, the result of which was 27/255 in the ACL repair group and 15/224 in the ACL reconstruction group (OR, 1.56; 95% CI, 0.81–3.00; *I*^2^ = 0%; *P* = 0.18) (Figure 3A).

A total of 6 studies (11, 12, 26, 28, 30, 32) reported postoperative complications, including superficial or deep infection, deep venous thrombosis (DVT), and pain at the tibial screw without the need for removal. There were 13/161 patients in the ACL repair group and 8/159 in the ACL reconstruction group (OR, 1.65; 95% CI, 0.65–4.15; *I*^2^ = 0%; *P* = 0.29) (Figure 3B).

Reoperations other than revision were defined as additional surgical interventions for ipsilateral knee disorders other than ACL retears, such as menisci lesions, synovitis, and arthrofibrosis. Reoperations were reported in 7 studies (11, 13, 14, 26, 30–32), and the result was 28/243 in the ACL repair group and 22/209 in the ACL reconstruction group (OR, 1.09; 95% CI, 0.60–1.99; *I*^2^ = 0%; *P* = 0.78) (Figure 3C).

Hardware removal rates were reported in 4 studies (11, 13, 26, 30), the result of which was 21/150 (19 in the DIS group, 2 in the BEAR group) in the ACL repair group and 3/119 (3 in the DIS group) in the ACL reconstruction group (OR, 6.84; 95% CI, 2.24–20.92; *I*^2^ = 0%; *P* = 0.0008) (Figure 3D).

### AP Knee Laxity

**Figure 4:**
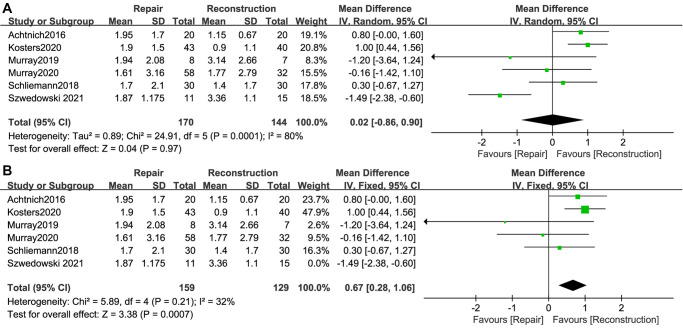
Meta-analysis of AP knee laxity (**A**) ΔATT; (**B**) ΔATT (sensitivity analysis).

A total of 6 studies (12–14, 26, 28, 32) reported ΔATT when stress was exerted on the knees, with a total of 170 cases in the ACL repair group and 144 cases in the ACL reconstruction group. The difference in postoperative ΔATT between the two groups was not statistically significant (WMD, 0.02; 95% CI, −0.86–0.90; *I*^2^ = 80%; *P* = 0.97) (Figure 4A). Because the *I*^2^ was relatively high, sensitivity analyses were performed by sequentially removing the included studies. After removing the study (28) by Szwedowski et al., the *I*^2^ decreased dramatically, and knees with ACL repair showed significantly greater ΔATT (WMD, 0.56; 95% CI, 0.04–1.08; *I*^2^ = 32%; *P* = 0.04) (Figure 4B).

### Patient-Reported Outcomes

**Figure 5:**
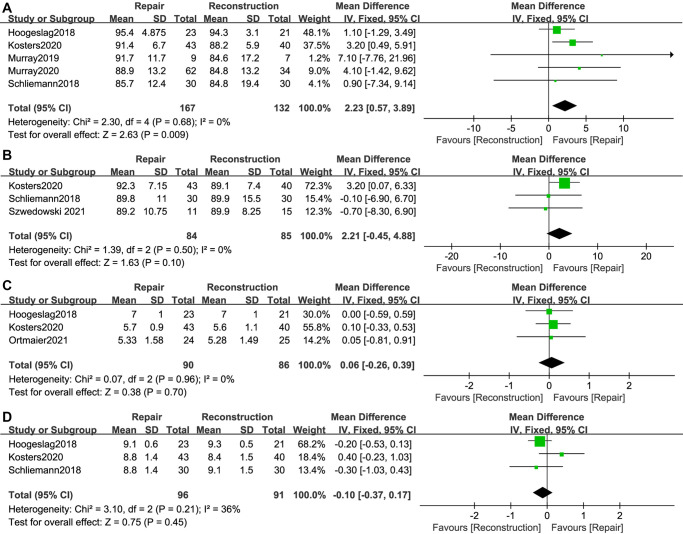
Meta-analysis of patient-reported outcomes (**A**) IKDC score; (**B**) Lysholm score; (**C**) Tegner score; (**D**) Satisfaction.

The IKDC score was mentioned in 5 studies (11–14, 26), with 167 cases in the ACL repair group and 132 cases in the ACL reconstruction group. ACL repair showed an advantage in terms of the postoperative IKDC score over ACL reconstruction (WMD, 2.23; 95% CI, 0.57–3.89; *I*^2^ = 0%; *P* = 0.009) (Figure 5A).

The Lysholm score was reported in 3 studies (12, 14, 28), involving 84 patients in the ACL repair group and 85 patients in the ACL reconstruction group. There was no significant difference in the postoperative Lysholm score between the two groups (WMD, 2.21; 95% CI, −0.45–4.88; *I*^2^ = 0%; *P* = 0.10) (Figure 5B).

Tegner score was reported in 3 studies (11, 12, 31). There were 90 cases in the ACL repair group and 86 cases in the ACL reconstruction group. Compared to ACL reconstruction, ACL repair provided comparable improvements in the postoperative Tegner score (WMD, 0.06; 95% CI, −0.26–0.39; *I*^2^ = 0%; *P* = 0.70) (Figure 5C).

A total of 3 studies (11, 12, 14) reported satisfaction (range, 0–10; 0 representing unsatisfied and 10 representing very satisfied) after surgery. With 96 patients in the ACL repair group and 91 patients in the ACL reconstruction group, no significant difference regarding patient satisfaction after surgery was found between the two groups (WMD, −0.10; 95% CI, −0.37–0.17; *I*^2 ^= 36%; *P* = 0.45) (Figure 5D).

### Subgroup Analyses

Subgroup analyses for the primary outcome failure rates according to the rupture location and ACL repair technique were performed. No significant differences between different repair techniques and rupture locations were observed (Table 3). Subgroup analyses according to RCT design or non-RCT design were also conducted for all outcomes including failure, complications, reoperation other than revision, hardware removal rates, AP Knee laxity and patient-reported outcomes, but no significant differences or changes of outcomes were found according to the designs (Table 4). All the important outcomes are listed in Table 5.

**Table 3:** Subgroup analysis of failure rates.

Variable	No. of studies	Failure rate		Risk ratio (95%CI)	*P* value
		Repair	Reconstruction		
ACL repair technique					
SAR	1	3/20	0/20	8.20 (0.40, 169.90)	0.17
BEAR	2	9/74	2/45	2.70 (0.55, 13.26)	0.22
IBLA	1	1/12	0/15	4.04 (0.15, 108.57)	0.41
DIS	4	14/149	13/144	1.02 (0.46, 2.29)	0.95
Main rupture location					
Independent	2	5/83	4/83	1.28 (0.32, 5.04)	0.73
Proximal	6	22/172	11/141	1.65 (0.78, 3.48)	0.19

*SAR, suture anchor repair; BEAR, bridge-enhanced ACL repair; IBLA, internal brace ligament augmentation; DIS, dynamic intraligamentary stabilization.*

**Table 4:** Subgroup analyses according to study design for all outcomes.

Outcomes	No. of studies	Study design		OR/WMD, (95%CI), *I*^2^		*P* value	
		RCT	Non-RCT	RCT	Non-RCT	RCT	Non-RCT
Failure	8	4	4	1.30, (0.57–2.92), 17%	2.17, (0.72–6.60), 0%	0.53	0.17
Complications	6	2	4	1.58, (0.47–5.33), 0%	1.75, (0.42–7.22), 0%	0.46	0.44
Reoperation other than revision	7	4	3	1.17, (0.52, 2.65), 0%	1.00, (0.41, 2.44), 0%	0.71	1.00
Hardware removal	4	2	2	1.68, (0.07, 42.27), NA	8.21, (2.48, 27.22), 0%	NA	0.0006
AP Knee laxity	6	3	3	0.55, (−0.13, 1.23), 43%	−0.54, (−2.34, 1.26), 86%	0.11	0.56
IKDC score	5	3	2	2.22, (0.51, 3.92), 0%	2.36, (−4.85, 9.56), 0%	0.01	0.52
Lysholm score	3	1	2	3.20, (0.07, 6.33), NA	−0.37, (−5.44, 4.70), 0%	NA	0.89
Tegner score	3	2	1	0.06, (−0.29, 0.42), 0%	0.05, (−0.81, 0.91), NA	0.72	NA
Satisfaction	3	3	0	−0.10, (−0.37, 0.17), 36%	NA	0.45	NA

*OR, odds ratio; WMD, weighted mean difference; CI, confidence interval; RCT, randomized controlled trial; NA, not applicable; AP, anteroposterior; IKDC, International Knee Documentation Committee.*

**Table 5:** Summary of findings table.

**Arthroscopic ACL repair for ACL ruptures**						
**Patient or population:** patients with ACL ruptures **Settings:** in skeletally mature patients **Intervention:** arthroscopic ACL repair **Comparison:** autograft ACL reconstruction						
**Outcomes**	**Illustrative comparative risks^a^** **(95% CI)**		**Relative effect** **(95% CI)**	**No of Participants** **(studies)**	**Quality of the evidence** **(GRADE)**	**Comments**
	Assumed risk	Corresponding risk				
	**Autograft ACL reconstruction**	**Arthroscopic ACL repair**
**Failure**	**Study population**		**OR 1.56** (0.81–3)	479 (8 studies)	⊕⊕⊕⊝ **moderate**	
	**67 per 1**,**000**	**101 per 1**,**000** (55–177)				
	**Moderate**					
	**29 per 1**,**000**	**45 per 1**,**000** (24–82)				
**Complication**	**Study population**		**OR 1.65** (0.65–4.15)	320 (6 studies)	⊕⊕⊕⊝ **moderate**	
	**50 per 1**,**000**	**80 per 1**,**000** (33–180)				
	**Moderate**					
	**22 per 1**,**000**	**36 per 1**,**000** (14–85)				
**Reoperation other than revision**	**Study population**		**OR 1.09** (0.6–1.99)	452 (7 studies)	⊕⊕⊕⊝ **moderate**	
	**105 per 1**,**000**	**114 per 1**,**000** (66–190)				
	**Moderate**					
	**100 per 1**,**000**	**108 per 1**,**000** (62–181)				
**Hardware removal**	**Study population**		**OR 6.84** (2.24–20.92)	269 (4 studies)	⊕⊕⊝⊝ **low**	
	**25 per 1**,**000**	**150 per 1**,**000** (55–351)				
	**Moderate**					
	**0 per 1**,**000**	**0 per 1**,**000** (0 to 0)				
**ΔATT**		The mean ΔATT in the intervention groups was **0.02 higher** (0.86 lower to 0.9 higher)		314 (6 studies)	⊕⊝⊝⊝ **very low**
**IKDC score**		The mean IKDC score in the intervention groups was **1.97 higher** (0.22–3.72 higher)		187 (3 studies)	⊕⊕⊝⊝ **low**
**Tegner score**		The mean Tegner score in the intervention groups was **0.06 higher** (0.26 lower to 0.39 higher)		176 (3 studies)	⊕⊕⊝⊝ **low**
**Lysholm score**		The mean Lysholm score in the intervention groups was **2.21 higher** (0.45 lower to 4.88 higher)		169 (3 studies)	⊕⊕⊝⊝ **low**
**Satisfaction**		The mean satisfaction in the intervention groups was **0.1 lower** (0.37 lower to 0.17 higher)		187 (3 studies)	⊕⊕⊕⊕ **high**

***CI,*** *Confidence interval; **OR,** Odds ratio.*

*GRADE Working Group grades of evidence.*

***High quality:*** *Further research is very unlikely to change our confidence in the estimate of effect.*

***Moderate quality:*** *Further research is likely to have an important impact on our confidence in the estimate of effect and may change the estimate.*

***Low quality:*** *Further research is very likely to have an important impact on our confidence in the estimate of effect and is likely to change the estimate.*

***Very low quality:*** *We are very uncertain about the estimate.*

*^a^* *The basis for the **assumed risk** (e.g. the median control group risk across studies) is provided in footnotes. The **corresponding risk** (and its 95% confidence interval) is based on the assumed risk in the comparison group and the **relative effect** of the intervention (and its 95% CI).*

## Discussion

This is the first meta-analysis to compare primary arthroscopic ACL repair to gold standard autograft ACL reconstruction. Different from previous results, this study revealed that, with a mean follow-up period from 12 to 36 months, patients who underwent arthroscopic ACL repair had statistically comparable outcomes of failure, complications, reoperation other than revision, Lysholm score, Tegner score, and satisfaction when compared with autograft ACL reconstruction. In addition, arthroscopic ACL repair showed subtle advantages in terms of the IKDC score but a higher rate of hardware removal and possibly greater ΔATT than ACL reconstruction.

ACL reconstruction has become the gold standard surgical treatment for ACL ruptures since the late 20th century. However, Van der List et al. (33) reviewed the evolutionary history of ACL surgical treatment modalities, and proposed that the paradigm shifting away from primary ACL repair was partly due to “unfortunate timing”. Immature arthroscopic technology, a long period of immobilization after surgery (34, 35), and an unbefitting choice of patients (1) contributed to poor outcomes following open ACL repair. In 1991, Sherman et al. (1) reported an important finding that patients with proximal tears and good tissue quality tended to have significantly better clinical results than those with other types of tears. In 1993, Genelin et al. (36) performed open repair for patients with proximal ACL tears, and observed excellent outcomes at mid-term follow-up. Several studies (37–39) had already reported similar favorable outcomes in the early 1980s. A systematic review by Van der List et al. (40) found that open primary ACL repair yielded excellent outcomes in patients with proximal tears. These results suggested that primary arthroscopic ACL repair might play a role in treating proximal ACL tears. Previous systematic reviews (6, 7, 9, 10) revealed that ACL reconstruction led to better survivorship and improvement in postoperative function than arthroscopic ACL repair, but the use of internal bracing, biological augmentation, and dynamic stabilization had the potential to increase the success rate of repair. With some newly published studies enrolled, this is the first review to report that arthroscopic ACL repair is not inferior to autograft ACL reconstruction, and even has some advantages over reconstruction.

In theory, primary arthroscopic ACL repair techniques have some advantages over autograft ACL reconstruction. For example, proprioception should be maintained (41), less invasive, early range of motion (ROM) regain (24), native kinematics should be restored (42), donor site morbidity should be avoided, and osteoarthritis should be prevented (43). In addition, standard ACL reconstruction can be easily performed if primary ACL repair fails, while revision surgery for failed ACL reconstruction is much more complex. Studies included in our meta-analysis showed outcomes in favor of these advantages. Vermeijden et al. (29) observed that patients who underwent ACL repair had less awareness of their operated knees than those who underwent reconstruction at the 2- to 5- year follow-up, especially in patients older than 30 years, male patients and patients with a body mass index larger than 25. Schliemann et al. (14) reported that there were no significant differences between the two groups for knee kinematic and kinetic parameters at any time of the 6-month follow-up, but those in the DIS group had better early activity at weeks 2 and 3 after surgery. Ortmaier et al. (31) concluded that patients with IBLA had the same return-to-sports activity level as patients with classic ACL reconstruction at short-term follow-up. However, whether these advantages can be maintained at longer follow-up is unclear, and needs to be verified by further studies.

In this meta-analysis, potential disadvantages of ACL repair were also observed. ACL repair showed a significantly higher rate of hardware removal, mainly due to more requests for implant removal in patients with DIS repair. In the DIS procedure, the monobloc spring- screw is much bulkier than that used in ACL reconstruction, because it has to withstand a high tensile load. Although it is unnecessary to remove the spring- screw when ACL heals, Henle et al. (44) reported that nearly half of patients asked to remove it without any clinical need, while this procedure is uncommonly performed after ACL reconstruction (45, 46). This might be a disadvantage of DIS, but the hardware removal procedure is a minimally invasive operation under local anesthesia, yielding almost no negative effects on the recovery process.

Another potential disadvantage of ACL repair is AP knee laxity. Inconsistent results were reported in the included studies. The overall pooled outcome suggested no significant difference between ACL repair and reconstruction, but the heterogeneity was considered to be high (*I*^2 ^= 80%). This heterogeneity might be caused by the differences in study design and ACL repair techniques. After removing the study by Szwedowski et al. (28), the *I*^2^ decreased to 32%, and ACL repair showed significantly larger ΔATT than reconstruction. These results indicated that arthroscopic ACL repair might be related to greater AP knee laxity than reconstruction, but this asymptomatic laxity can only be observed when stress is exerted on the knees. Further studies should pay attention to long-term postoperative AP knee laxity in the two groups.

To further explore potential factors that would influence failure rates, we performed subgroup analyses. The rupture location and ACL repair technique are essential. Among the 10 included studies, 8 studies (11–13, 26, 28, 29, 31, 32) mainly enrolled patients with proximal ruptures, and the other 2 studies (14, 30) enrolled patients regardless of the rupture location. For ACL repair techniques, 1 study (32) used SAR, 2 studies (13, 26) used BEAR, 3 studies (28, 29, 31) used IBLA, and 4 studies (11, 12, 14, 30) used DIS. After subgroup analyses according to the rupture location and repair technique, no significant differences between different rupture locations and repair techniques were observed. However, these results should be interpreted with caution because the number of studies that enrolled patients regardless of the rupture location was only 2, and the number of studies using SAR and BEAR was also limited.

This study is not without limitation. First, although we enrolled more comparative studies than any other previous systematic review, the number of RCTs was only 4, and the majority of included studies were cohort and case–control studies. Second, due to a short history of arthroscopic ACL repair, the mean follow-up period of the included studies was not long enough (12–36 months). Third, the ACL repair techniques are varied, and differences in detailed surgical methods and the type of graft for ACL reconstruction were inevitable. However, the biomechanical effect of ACL reconstructions could be considered to be approximately equivalent. Fourth, we failed to conduct a meta-analysis of radiological results after surgery. Although good radiological results were observed, only 2 studies reported ACL healing examined by MRI and 1 study reported signs of osteoarthritis detected by X-rays at the 2-year follow up. Last, rehabilitation courses after surgery varied among studies, which could have influenced postoperative outcomes.

## Conclusion

Compared to the autograft ACL reconstruction, arthroscopic ACL repair showed similar clinical outcomes, and even better functional performance in treating proximal ACL ruptures. ACL repair has a higher rate of hardware removal, and might be related to greater asymptomatic knee laxity. Arthroscopic ACL repair might be a viable option for patients with proximal ACL ruptures. However, limited by the amount of high-quality evidence (only 4 RCTs), we cannot draw a definite conclusion. More high-quality prospective trials comparing these two techniques are needed to confirm our findings.

## Data Availability

The original contributions presented in the study are included in the article/supplementary material, further inquiries can be directed to the corresponding author/s.
